# A pH-responsive soluble polymer-based homogeneous system for fast and highly efficient *N*-glycoprotein/glycopeptide enrichment and identification by mass spectrometry†
†Electronic supplementary information (ESI) available. See DOI: 10.1039/c5sc00396b



**DOI:** 10.1039/c5sc00396b

**Published:** 2015-05-26

**Authors:** Haihong Bai, Chao Fan, Wanjun Zhang, Yiting Pan, Lin Ma, Wantao Ying, Jianhua Wang, Yulin Deng, Xiaohong Qian, Weijie Qin

**Affiliations:** a National Center for Protein Sciences Beijing , State Key Laboratory of Proteomics , Beijing Proteome Research Center , Tianjin Baodi Hospital , Beijing Institute of Radiation Medicine , China . Email: aunp_dna@126.com ; Email: qianxh1@163.com; b School of Life Science and Technology , Beijing Institute of Technology , Beijing , China; c Research Center for Analytical Sciences , College of Sciences , Northeastern University , Shenyang , China

## Abstract

Liquid phase homogeneous reactions using soluble polymer supports have found numerous applications in homogeneous catalysis and organic synthesis because of their advantages of no interface mass transfer limitation and a high conversion rate. However, their application in analytical separation is limited by the inefficient/inconvenient recovery of the target molecules from the extremely complex biological samples. Here, we report a stimuli-responsive polymer system for facile and efficient enrichment of trace amounts of biomolecules from complex biological samples. The soluble polymer supports provide a homogeneous reaction system with fast mass transfer and facilitate interactions between the supports and the target molecules. More importantly, the stimuli-responsive polymers exhibit reversible self-assembly and phase separation under pH variations, which leads to facial sample recovery with a high yield of the target biomolecules. The stimuli-responsive polymer is successfully applied to the enrichment of low abundant *N*-glycoproteins/glycopeptides, which play crucial roles in various key biological processes in mammals and are closely correlated with the occurrence, progression and metastasis of cancer. *N*-Glycoprotein is coupled to the stimuli-responsive polymer using the reported hydrazide chemistry with pre-oxidation of the oligosaccharide structure. Highly efficient enrichment of *N*-glycoproteins/*N*-glycopeptides with >95% conversion rate is achieved within 1 h, which is eight times faster than using solid/insoluble hydrazide enrichment materials. Mass spectrometry analysis achieves low femtomolar identification sensitivity and obtained 1317 *N*-glycopeptides corresponding to 458 *N*-glycoproteins in mouse brain, which is more than twice the amount obtained after enrichment using commercial solid/insoluble materials. These results demonstrate the capability of this “smart” polymer system to combine stimuli-responsive and target-enrichment moieties to achieve improved identification of key biological and disease related biomolecules.

## Abstract 2

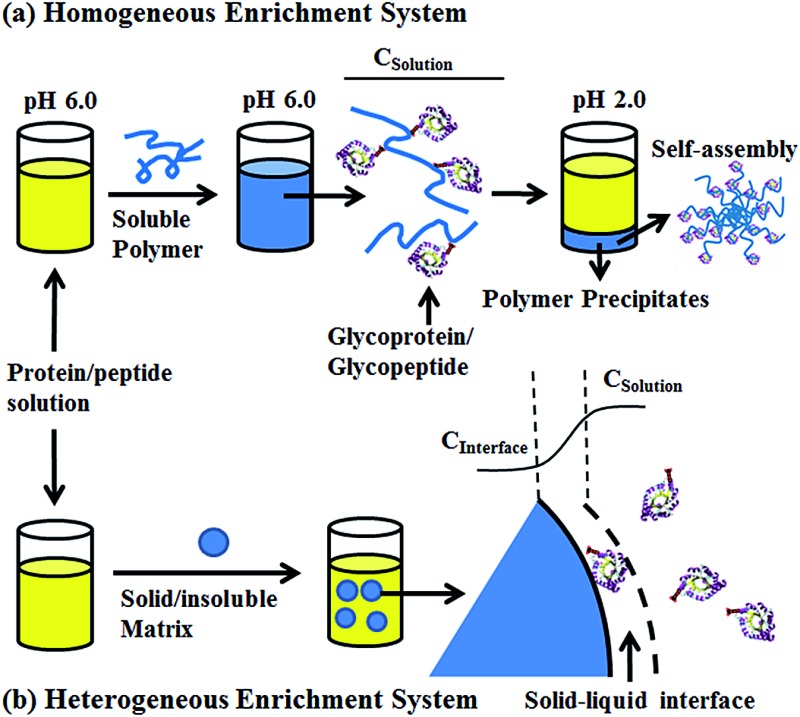
A homogeneous reaction system was developed for facile and highly efficient enrichment of biomolecules by exploiting the reversible self-assembly of a stimuli-responsive polymer.

## Introduction

Stimuli-responsive polymers or “smart” polymers exhibit predictable and sharp changes in properties in response to small environmental changes, such as temperature, pH, ionic strength, light, or mechanical stress. These changes cause reversible self-assembly or phase separation of the polymer, which have attracted significant research attention in the areas of synthesis of switchable pores/surfaces, biomedical imaging/diagnostics and controlled drug delivery.^1–6^ However, the potential of these “smart” polymers has not been well explored in the field of analytical separation, which is extremely important for the sensitive identification and quantification of biomolecules and chemical components in biological, pharmaceutical and environmental analysis. Currently, the lack of fast and highly efficient sample enrichment/separation methods remains a major obstacle for achieving high throughput and sensitive analysis.^7,8^ This lack of methodology is especially the case for biological analysis, due to the limited sample amount, low target concentration and strong background interference.^9,10^ The widely adopted solid/insoluble matrix based enrichment approaches suffer from large steric hindrance, heterogeneous reactions between the liquid-phase targets and the solid-phase ligand and diffusion limitation in the solid–liquid interface.^11,12^ These unfavorable conditions may result in limited reaction rates and poor yields. In contrast, homogeneous reactions in the liquid phase have the obvious advantages of fast mass transfer and high conversion rates and have been demonstrated to be highly useful in homogeneous catalysis and liquid-phase synthesis.^13,14^ However, the lack of facile and robust target recovery approaches prevents the adoption of this approach in biological analysis, which commonly involves very small amounts of samples. Therefore, a new enrichment matrix with homogeneous reaction nature, improved accessibility and facile recovery is in urgent need for biological analysis.

As one of the most important post-translational modifications^15–17^ in eukaryote cells and key bio-analytical targets, protein *N*-glycosylation plays crucial roles in various biological processes, including intercellular recognition and communication, protein folding and immune responses.^18–20^ Aberrant protein *N*-glycosylation is closely associated with many major human diseases, such as inflammation, metabolic disorders, and various types of cancers, offering promising potential for disease diagnosis or prognostic monitoring.^21–24^ Sensitive identification of these disease-related *N*-glycosylation variations may provide a unique path for the development of new diagnostics biomarkers and therapeutic drug targets.^25–27^ Shotgun based glycoproteomics analysis by mass spectrometry (MS) is currently the method of choice for large scale and in-depth glycoprotein/glycopeptide profiling in complex biological samples.^28,29^ However, the inherently low abundant glycopeptides (approximately 1–2% of the total amount of peptides) obtained from tryptic digest of complex protein samples makes glycopeptide enrichment a prerequisite for efficient identification. Although a variety of enrichment methods such as hydrazide chemistry,^30–32^ boronic acid,^33–35^ lectin affinity^36^ and HILIC^37,38^ have been developed, the enormous complexity of the biological samples makes comprehensive enrichment and sensitive identification of glycopeptides by mass spectrometry still a challenging task. Despite varying mechanisms, most of the reported glycopeptides enrichment methods rely on the solid–liquid heterogeneous reaction using solid/insoluble matrix. The disadvantages of the interfacial mass transfer resistance and nonlinear kinetic behaviour of this reaction system as well as the high steric hindrance of the matrix materials are the major obstacles limiting the reaction rate and enrichment efficiency.

**Scheme 1:**
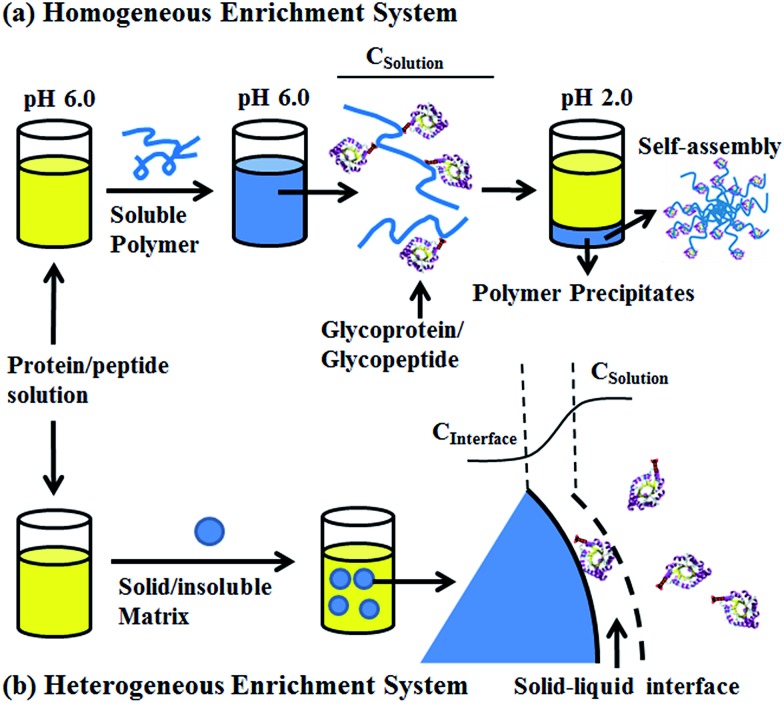
Schematic overview of the soluble polymer (a), solid/insoluble matrix (b) based glycoproteins/glycopeptides enrichment. *C*_Solution_: protein or peptide concentration in the bulk solution, *C*_Interface_: protein or peptide concentration at the solid–liquid interface.

To solve this problem, we developed a pH-responsive soluble enrichment matrix that can be reversibly dissolved and self-assembled in aqueous solution for homogeneous reaction-based enrichment (Scheme 1). The soluble enrichment matrix is prepared by copolymerization of pH-responsive monomers with glycan-reactive moieties. The obtained linear copolymer chains form a homogeneous reaction mixture with the protein/peptides samples in aqueous solution under mildly acidic pH which facilitates the coupling between the polymer matrix and the target glycoprotein/glycopeptides. Facile sample recovery with high efficiency can be achieved by simply lowering the system pH, which results in the rapid self-assembly of the polymer–glycoprotein/glycopeptide conjugates into large particle agglomerates that precipitate from the solution. Therefore, only a single-step solution-phase reaction is involved in the enrichment process. Compared with the conventional solid/insoluble enrichment matrix, this stimuli-responsive polymer-based soluble enrichment matrix has three advantages. First, fast enrichment with >95% efficiency can be achieved within 1 h due to the unrestricted mass transfer in the homogeneous enrichment system. Second, due to the densely packed accessible glycan-reactive moieties on the linear polymer chains and pH-responsive-based facile recovery, advances in the enrichment of trace amount of glycoprotein/glycopeptides in complex biological samples can be expected. Finally, the substantially reduced steric hindrance caused by applying flexible linear polymer chains instead of beads/particles as the enrichment matrix may facilitate the capturing and subsequent enzymatic releasing of the target glycopeptides. As a result, we expect that this new enrichment matrix can be applied as a general approach to promote biomolecule identification.

## Results and discussion

### Synthesis and characterization of poly-(AA-*co*-hydrazide)

**Scheme 2:**
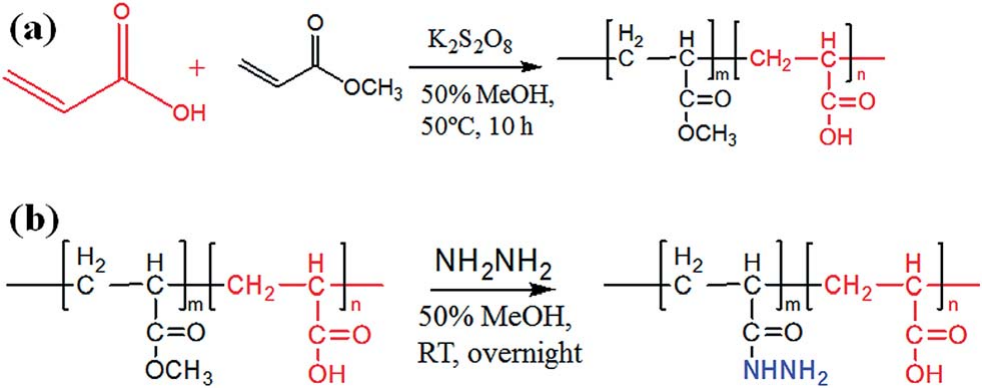
Synthesis of poly-(AA-*co*-MA) copolymer and hydrazide functionalization.

**Fig. 1:**
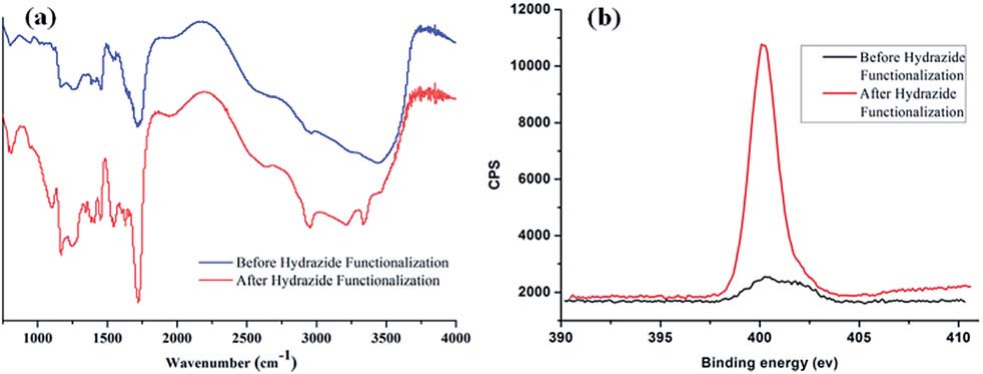
FTIR and XPS characterization of poly-(AA-*co*-MA) copolymer before and after hydrazide modification.

The preparation of poly-(acrylic acid) based pH-responsive polymer with hydrazide functionalization for glycoprotein/glycopeptide enrichment is shown in Scheme 2. First, poly-(acrylic acid-*co*-methyl acrylate) was synthesized by copolymerization of acrylic acid (AA) and methyl acrylate (MA). GPC analysis of the obtained poly-(AA-*co*-MA) copolymer with 1–20 h polymerization time reveals molecular weights ranging from *M*_n_ = 15 440 to 214 100 g mol^–1^ and *M*_w_/*M*_n_ = 1.183–1.702 (Table S1†). Next, the pH response of poly-(AA-*co*-MA) of different molecular weights was evaluated. Strong pH response was observed for the soluble polymer with molecular weight ∼150 kg mol^–1^ or higher (Fig. S1†), as the clear polymer solution immediately turns to a milky white suspension after changing the pH from 6.0 to 2.0. Next, the methyl ester in the poly-(AA-*co*-MA) copolymer was converted to hydrazide by hydrazine monohydrate treatment. The poly-(AA-*co*-MA) copolymer before and after hydrazine treatment was characterized by FTIR (Fig. 1a) and XPS (Fig. 1b). The successful synthesis of poly-(AA-*co*-MA) copolymer was confirmed by the characteristic peaks at 1730 cm^–1^ (C=O stretching of the carbonyl), 1385 cm^–1^ (COO symmetric stretching of the carboxylate ion), 1255 cm^–1^ (C–O stretching of the COOH group) and 1170 cm^–1^ (antisymmetric stretching of the C–O–C of ester groups) in the FTIR spectrum. After hydrazine treatment, new peaks appeared at approximately 1630 cm^–1^ (N–H bending), 3210 cm^–1^, 3350 cm^–1^ (N–H stretching) and 1405 cm^–1^ (C–N stretching), indicating the introduction of C–NH–NH_2_ groups in the copolymer chain. The conversion of the methyl ester of poly-(AA-*co*-MA) to hydrazide was further demonstrated by X-ray photoelectron spectroscopy (XPS) analysis. As shown in Fig. 1b, the introduction of hydrazide to poly-(AA-*co*-MA) leads to a clear exhibition of the N 1s peak at 400.1 eV in the XPS spectrum. In contrast, no corresponding peak can be found for the untreated poly-(AA-*co*-MA). Next, the hydrazide loading of the poly-(AA-*co*-hydrazide) was determined using the bicinchoninic acid (BCA) titrating method, which relies on the reduction of Cu^2+^ to Cu^+^ by hydrazide and the colorimetric detection of the Cu^1+^ by bicinchoninic acid at 562 nm. The standard curve of the BCA titration of poly-(AA-*co*-hydrazide) copolymer is shown in Fig. S2† and the hydrazide loading was found to be 1.2 mmol g^–1^, which is more than six times higher than that of the commercial cross-linked agarose–hydrazide beads. The obviously increased hydrazide loading of poly-(AA-*co*-hydrazide) can be explained by the fact that all of the methyl ester groups of the linear polymer chains of poly-(AA-*co*-MA) are theoretically accessible for hydrazide functionalization. In contrast, for solid materials, such as cross-linked agarose beads (50–150 μm in diameter), only the molecules at the surface layer of the beads are exposed and the inner molecules are inaccessible for functionalization. The high hydrazide density of poly-(AA-*co*-hydrazide) can increase its collision opportunity and binding capacity with glycoproteins in complex protein samples, therefore it is particularly advantageous for glycosylation identification.

### The pH-induced self-assembly of poly-(AA-*co*-hydrazide)–glycoprotein conjugates

**Fig. 2:**
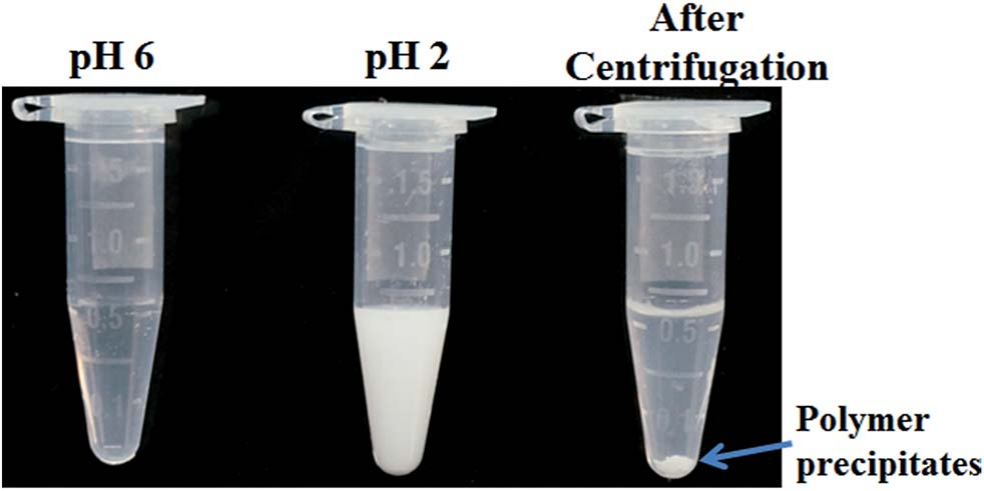
Dispersity analysis of poly-(AA-*co*-hydrazide)–RNase B conjugates at pH 6.0 and 2.0.

**Fig. 3:**
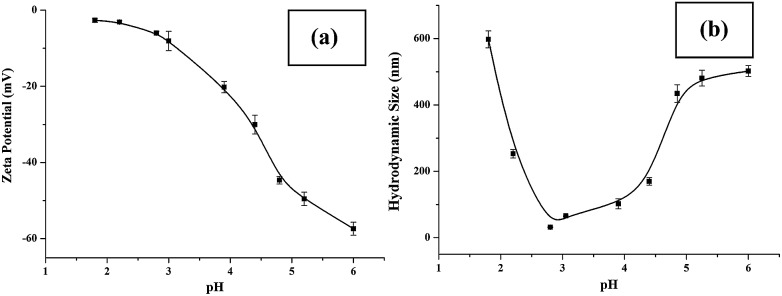
Zeta potential (a) and hydrodynamic size (b) of the poly-(AA-*co*-hydrazide)–RNase B conjugates at different pH values. Results are expressed as the mean of three separated measurements ± standard deviation (SD).

Sensitive pH-response of the soluble polymer after conjugation with target molecules is a prerequisite for achieving high sample recovery in enrichment reactions. Therefore, the pH-responsive behavior of poly-(AA-*co*-hydrazide) after conjugation with a standard glycoprotein (RNase B) was analyzed using solutions with varying pH values to evaluate the feasibility of this strategy for glycoprotein enrichment. We found that the poly-(AA-*co*-hydrazide)–RNase B conjugates dissolve well in aqueous solution under neutral to mildly acidic pH and rapidly precipitate in an acidic environment. As shown in Fig. 2, the clear solution immediately turns to a milky white suspension after changing the pH from 6.0 to 2.0 due to the large scale self-assembly of the conjugates. White polymer precipitates can be easily collected by gentle centrifugation for a few seconds. These results demonstrate that the conjugation of glycoproteins does not interfere with the pH-induced aggregation of the poly-(AA-*co*-hydrazide) copolymer chains. To further investigate the pH dependent self-assembly and aggregation of the poly-(AA-*co*-hydrazide)–glycoprotein conjugates, the zeta potentials and hydrodynamic size of the conjugates under different pH conditions were measured by dynamic light scattering (DLS). The zeta potential shows a monotonic increasing trend as the pH decreases over the range from 6 to 2, which can be explained by the gradual protonation of the acrylic acid moieties of the copolymer and the continuous shielding of the electrostatic charges (Fig. 3a). In the hydrodynamic size analysis (Fig. 3b), the conjugates exhibit decreasing hydrodynamic sizes as the pH decreases over the range from 6 to 2.8 because of the pH-induced shrinking of the copolymer chains. The minimal hydrodynamic size of ∼30 nm is reached at pH 2.8 and the corresponding zeta potential is –6.04. Further pH reduction by 0.6 leads to only a ∼3 mV increase in zeta potential but an abrupt increase in hydrodynamic size by approximately eight-fold to ∼250 nm. This result can be attributed to the large scale self-assembly and aggregation of the poly-(AA-*co*-hydrazide)–RNase B conjugates because the polymer micelles are highly unstable when the zeta potentials are over the range of 0 ± 5 mV. The results of the zeta potential and hydrodynamic size analysis demonstrate that the sensitive pH response of poly-(AA-*co*-hydrazide) is not impaired after glycoprotein conjugation. Furthermore, the pH-induced transformation of the well dispersed poly-(AA-*co*-hydrazide)–glycoprotein conjugates into aggregated clusters of approximately sub-micrometer size serves the purpose of enrichment very well. Because repeated precipitation–dissolution cycles are commonly involved in the enrichment process to remove the non-specifically adsorbed protein/peptides, the reproducibility of the pH responsive behavior of the poly-(AA-*co*-hydrazide)–glycoprotein conjugates was investigated. As shown in Fig. S3,† the transparency of the solution containing dissolved or self-assembled conjugates was obtained by UV adsorption analysis. No obvious transparency change was found after eight precipitation–dissolution cycles indicating the unimpaired pH responsiveness and robustness of this enrichment approach.

### Reaction kinetics, efficiency and selectivity of glycoprotein/glycopeptide enrichment using poly-(AA-*co*-hydrazide)

**Fig. 4:**
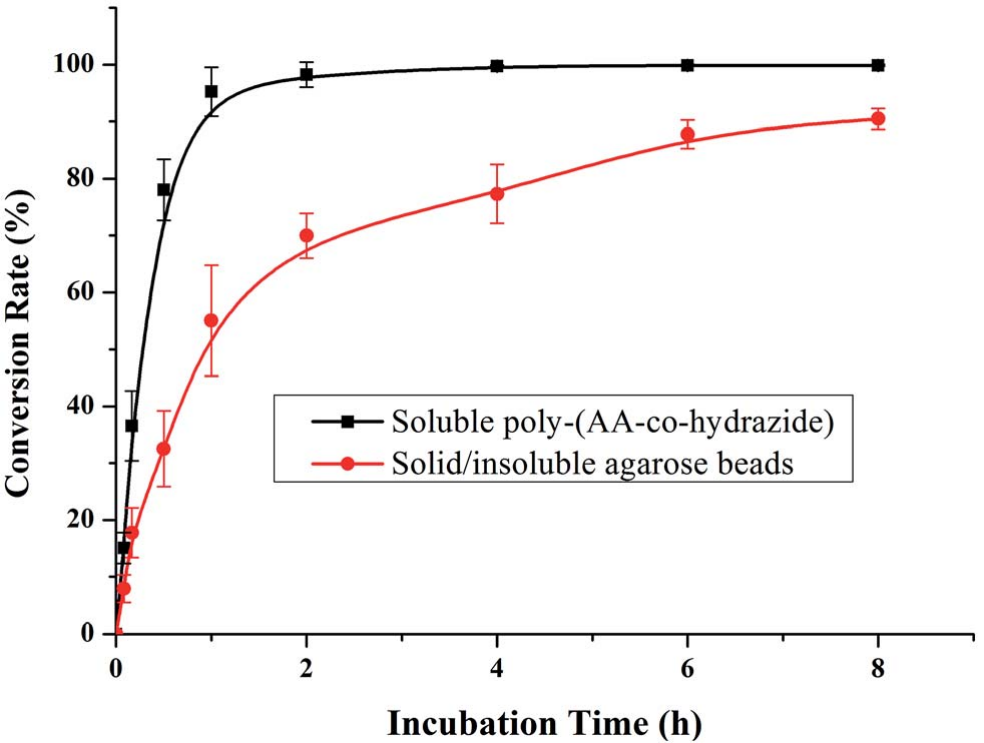
Kinetics analysis of glycoprotein enrichment using soluble poly-(AA-*co*-hydrazide) or solid/insoluble agarose beads. Results are expressed as the mean of three separated measurements ± standard deviation (SD).

After evaluating the pH responsiveness of the poly-(AA-*co*-hydrazide)–glycoprotein conjugates, the reaction kinetics, the conversion rate of aldehyde–hydrazide coupling and the recovery of glycoprotein enrichment using this soluble polymer matrix were investigated. First, the oligosaccharide structure of the *N*-glycoprotein was oxidized to produce aldehyde. Next, for the solid/insoluble enrichment matrixes (beads/particles), overnight incubation with the protein sample is required for complete glycoprotein enrichment^27^ due to the limited mass transfer in the liquid–solid interface in the heterogeneous reaction system and the large steric hindrance induced by the solid enrichment matrix. To demonstrate the advantages of using the soluble polymer-based homogeneous enrichment system, the reaction kinetics and the recovery of glycoprotein enrichment were studied using asialofetuin (a standard glycoprotein) and were compared with the results obtained using commercial solid agarose–hydrazide beads. As shown in Fig. 4, poly-(AA-*co*-hydrazide) has a faster reaction rate with the glycoprotein than solid agarose–hydrazide beads and 96.2% glycoprotein capturing is achieved *via* aldehyde–hydrazide coupling in 1 h. In contrast, only 55.6% enrichment conversion is reached after 1 h and at least 8 h of reaction are required to achieve >90% using the solid agarose–hydrazide beads. The recovery of glycoprotein enrichment using soluble poly-(AA-*co*-hydrazide) was determined after collecting the poly-(AA-*co*-hydrazide)–asialofetuin conjugates *via* low pH-induced precipitation. The obtained conjugates were treated with PNGAse F to cleave the covalent bond between the innermost GlcNAc of the *N*-linked glycans coupled with poly-(AA-*co*-hydrazide) and the asparagine residues of the glycoproteins. Subsequently, the released asialofetuin was further characterized and quantified by SDS-PAGE and ∼90% sample recovery was achieved (Fig. S4†). The improved reaction rate, efficient glycoprotein coupling and high recovery may be attributed to the unrestricted mass transfer in the homogeneous reaction based enrichment and the reduced steric hindrance using linear soluble poly-(AA-*co*-hydrazide) as the matrix. Improved accessibility of PNGase F towards the enzymatic cleavage site between the *N*-glycans and peptides can be expected. The facilitated release of the peptides may lead to enhanced detection in MS.

**Fig. 5:**
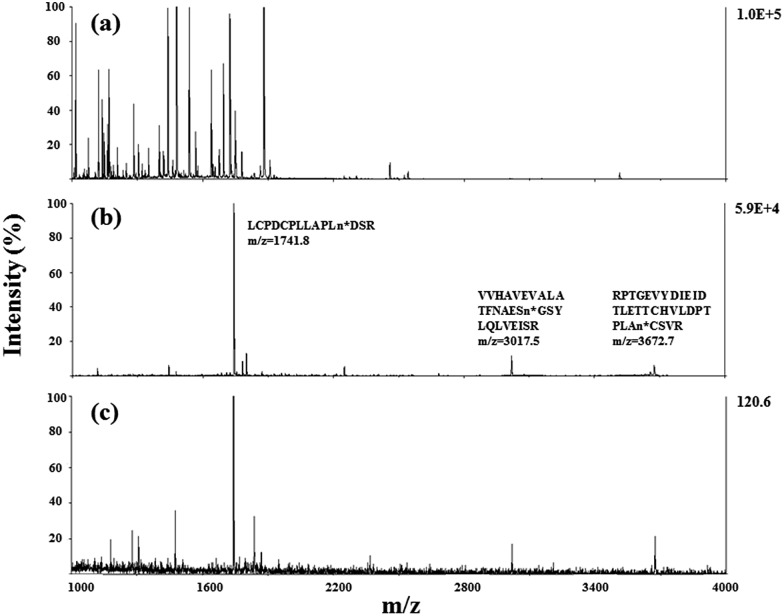
MALDI-TOF-MS analysis of the tryptic digests of the mixture of asialofetuin and BSA at 1 : 100 molar ratio. Asialofetuin (100 fmol) before (a) and after (b) enrichment using poly-(AA-*co*-hydrazide). Asialofetuin (1 fmol) after enrichment (c).

**Fig. 6:**
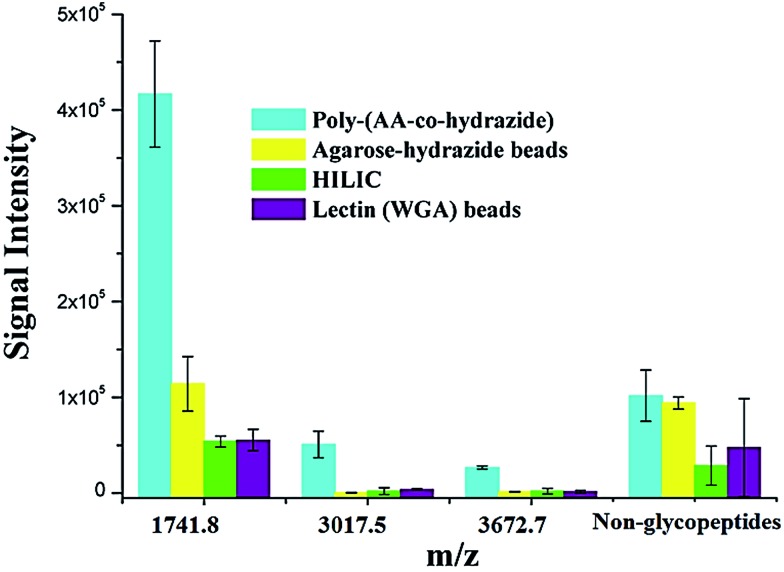
Comparison of the MALDI-TOF-MS signal intensities of the enriched *N*-glycopeptides of asialofetuin obtained using different enrichment approaches. Results are expressed as the mean of three separated measurements ± standard deviation (SD). Sequence and modification site of the identified *N*-glycopeptides: *m*/*z* = 1741.8 LCPDCPLLAPLn*DSR, *m*/*z* = 3017.5 VVHAVEVALATFNAESn*GSYLQLVEISR, *m*/*z* = 3672.7 RPTGEVYDIEIDTLETTCHVLDPTPLAn*CSVR. For the non-glycopeptides, the sum of the intensity of all the non-glycosylated peptide residues in the MS spectrum was used.

Due to the low abundance of glycopeptides in real biological samples, the ability to selectively enrich highly diluted glycopeptides from a complex solution is a key evaluating criterion for the soluble polymer based homogeneous system. A mixture of BSA (a non-glycoprotein) and asialofetuin (a standard glycoprotein with three well-characterized *N*-glycosylation sites) was used to mimic a complex sample. Fig. 5 shows the MALDI-TOF-MS spectra of the mixture of tryptic digests of 100 fmol asialofetuin and 10 pmol BSA before (a) and after (b) enrichment by poly-(AA-*co*-hydrazide). Before enrichment, the spectrum is overwhelmed by the signals of the abundant non-glycopeptides and the glycopeptides can be hardly detected due to the strong signal suppression by the non-glycopeptides (Fig. 5a). In contrast, nearly all of the non-glycopeptides are removed and three glycopeptides covering all of asialofetuin's theoretical glycosites are clearly detected with high signal intensities and *S*/*N* ratios after enrichment by poly-(AA-*co*-hydrazide) (Fig. 5b). For example, the signal intensity and *S*/*N* of the glycopeptide at 1741.8 *m*/*z* increases 29 and 325 times after enrichment, respectively. These results indicate the excellent selectivity of this method because only negligible non-glycopeptides remain after enrichment, although their concentration was one hundred times higher than that of the glycopeptides. Further diluting the amount of asialofetuin to 1 fmol while maintaining the same molar ratio between asialofetuin and BSA still results in the successful identification of the three glycopeptides (Fig. 5c), demonstrating that high enrichment efficiency and low fmol detection sensitivity can be reached after enrichment using the soluble polymer-based enrichment system. The advantages of using poly-(AA-*co*-hydrazide) were further demonstrated by a comparison with three other commonly used glycopeptide enrichment materials. Fig. 6 shows the MALDI-TOF-MS signal intensity of the enriched glycopeptides of asialofetuin by poly-(AA-*co*-hydrazide), by cross-linked agarose–hydrazide beads, by commercial HILIC materials and by agarose bead-bound lectins (WGA). Clearly, poly-(AA-*co*-hydrazide) provides the strongest signal intensity among the four enrichment materials for all of the three glycopeptides. The other three enrichment materials only resulted in a minor enhancement in the signal intensity of the glycopeptides, presumably due to the relatively lower enrichment affinity/selectivity or the unfavorable reaction conditions in the solid/insoluble matrix-based enrichment.

### Application of poly-(AA-*co*-hydrazide) for mouse brain glycopeptide enrichment and glycoprotein identification by mass spectrometry

**Fig. 7:**
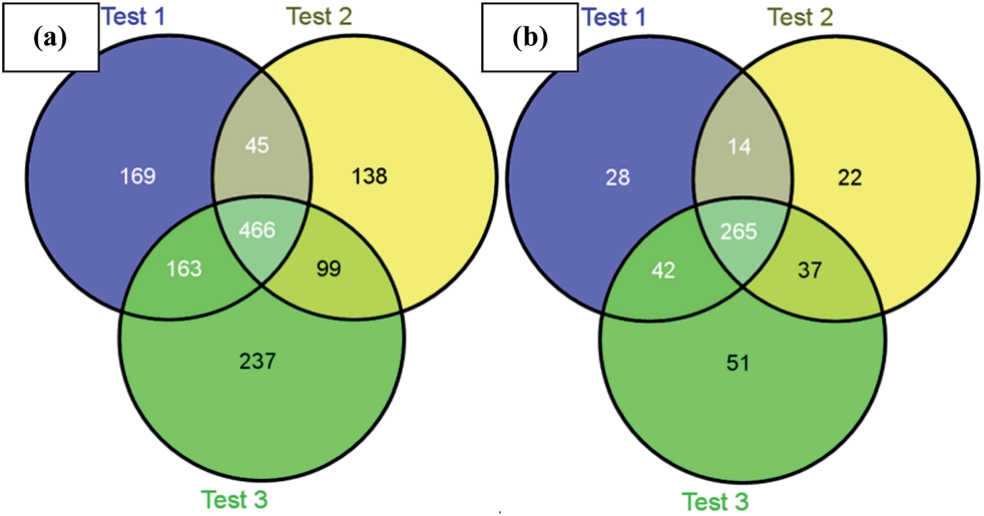
Identified mouse brain *N*-glycopeptides (a) and *N*-glycoproteins (b) using poly-(AA-*co*-hydrazide) based enrichment.

Next, we challenged the enrichment capability of poly-(AA-*co*-hydrazide) using highly complex protein extracts from mouse brain. The oxidized *N*-glycoproteins were first coupled with poly-(AA-*co*-hydrazide). After repeated washing to remove non-glycoproteins, the poly-(AA-*co*-hydrazide)–glycoprotein conjugates were subjected to trypsin digestion and repeated washing to remove non-glycopeptides. Next, the obtained poly-(AA-*co*-hydrazide)–glycopeptide conjugates were treated with PNGase F to release the enriched *N*-glycopeptides for LC-MS analysis by a LTQ-FT mass spectrometer. In three replicates, 843, 748 and 965 *N*-glycopeptides and 349, 338 and 395 *N*-glycoproteins were identified, corresponding to 1317 non-redundant *N*-glycopeptides and 458 non-redundant glycoproteins (Table S2†). As shown in Fig. 7, 80.0% of *N*-glycoproteins were identified in at least two replicates, demonstrating good reproducibility of this enrichment method. Compared with 56 or 533 non-redundant *N*-glycopeptides identified from triplicate experiments without enrichment or enrichment using commercial cross-linked agarose–hydrazide beads (Table S3†), the poly-(AA-*co*-hydrazide)-based soluble enrichment matrix shows obvious advantages for larger scale protein glycosylation identification. We attributed the improved glycopeptide enrichment to the high hydrazide density of poly-(AA-*co*-hydrazide) and the favorable mass transfer in the homogeneous hydrazide–aldehyde coupling reaction, which facilitate the collisions between poly-(AA-*co*-hydrazide) and the target glycopeptides. Furthermore, the substantially reduced steric hindrance caused by replacing the bulky solid/insoluble enrichment matrix with the flexible soluble polymer is particularly beneficial for enhancing the accessibility of the enzymatic cleavage sites of the matrix-immobilized glycopeptides. Therefore, highly efficient PNGase F enzymatic release of the captured glycopeptides can be expected.

Finally, to evaluate the reliability of the *N*-glycosylation identification obtained by the poly-(AA-*co*-hydrazide)-based enrichment, the spontaneous deamidation caused false-discovery of *N*-glycopeptides in this approach was determined. Control experiments using conditions identical to those used in the poly-(AA-*co*-hydrazide)-based glycopeptides enrichment and LC-MS analysis were conducted, except that the enriched peptides were not treated with PNGase F. Therefore, peptides with a 0.984 Da shift at Asn and the N-X-S/T/C (X ≠ P) motif identified in this experiment are the falsely discovered *N*-glycopeptides. In three replicates, we found 21 falsely discovered *N*-glycopeptides, corresponding to a 1.46% average false-discovery rate. This result is consistent with the literature reported value^39^ suggesting that spontaneous deamidation is a minor issue in the poly-(AA-*co*-hydrazide)-based glycopeptides enrichment. Thus, this false discovery will not jeopardize the reliability of the *N*-glycosylation assignment in this research.

## Conclusions

In conclusion, we developed a robust soluble polymer-based homogeneous enrichment system using pH-responsive poly-(AA-*co*-hydrazide). Improved enrichment reaction kinetics and enrichment efficiency of glycopeptides are achieved for standard protein and complex protein samples from animal tissue. We expect that this new enrichment approach will be widely applicable for the efficient enrichment of trace amounts of biomolecules and therefore promote bio-analysis.

## Experimental

### Materials and reagents

Bovine serum albumin (BSA), RNase B, bovine asialofetuin, acrylic acid, methyl acrylate and potassium persulfate were obtained from Sigma (St. Louis, MO, USA). Hydrazine monohydrate was purchased from Alfa Aesar (Ward Hill, MA, USA). Agarose-bound lectins (WGA) were purchased from Vector Labs (Burlingame, CA, USA), cross-linked agarose–hydrazide beads were acquired from GE Healthcare (Pittsburgh, PA, USA) and ZIC-HILIC beads (50 μm, 60 Å) were obtained from Merck (Darmstadt, Germany). Sequencing grade porcine trypsin was received from Promega (Madison, WI, USA). PNGase F was obtained from New England Biolabs (Beverly, MA, USA). Mice (C57BL/6J, males, aged 9–13 weeks) were received from Beijing HFK Bioscience company (Beijing, China). The deionized water used in all of the experiments (resistance > 18 MΩ cm^–1^) was prepared using a Millipore purification system (Billerica, MA, USA).

### Synthesis of pH-responsive acrylic acid–methyl acrylate copolymer (PAA-*co*-PMA) and hydrazide functionalization

Methyl acrylate (260 mg), 1730 mg of acrylic acid (MA : AA molar ratio of 1 : 8) and 50 mg of potassium persulfate were dissolved in 50 mL of degassed 50% methanol. The mixture was allowed to react in a nitrogen environment at 50 °C for 1–20 h with vigorous stirring. The obtained poly-(AA-*co*-MA) polymer was precipitated and recovered by the addition of pure ethanol. Next, the purified poly-(AA-*co*-MA) was re-dispersed in 50% methanol. The methyl ester groups of the copolymer were converted to hydrazide by the addition of 300 mg hydrazine monohydrate and the reaction was allowed to proceed overnight at RT under stirring. After removing the solvent by rotary evaporation and removing the residual reactants by dialysis (3000 cut-off), the obtained poly-(AA-*co*-hydrazide) was lyophilized and stored at 4 °C until further use. Before application in the *N*-glycoproteome enrichment, the poly-(AA-*co*-hydrazide) was first subjected to three times repeat washing by the low-pH-precipitation and high-pH-dissolution cycle to remove any trace amount of polymer chains with poor pH response.

### Characterization

Gel permeation chromatography (GPC) analysis was performed on a DAWN HELEOS system (Wyatt Technology, Santa Barbara, CA, USA). FTIR measurement was conducted in transmission mode using a FTS135 FTIR spectrophotometer (Bio-Rad, Hercules, CA, USA) under ambient conditions. All of the samples were ground and mixed with KBr and pressed to form pellets. The X-ray photoelectron spectroscopy (XPS) measurement was performed using a Kratos AMICUS system (Shimadzu, Japan) with Mg KR radiation (*hν* = 12 kV) at a power of 180 W. The hydrodynamic size was measured by dynamic light scattering (DLS) at 25 °C using a Zetasizer Nano ZS (Malvern Instruments, Worcestershire, UK). The excitation light source was a 4 mW He–Ne laser at 633 nm and the intensity of the scattered light was measured at 173°.

### Protein extraction

All the animal experiments were performed in compliance with the relevant regulations of Beijing Proteome Research Center (BPRC) and were approved by the "Committee for Animal Experiments" of BPRC. Mouse brain tissue was taken out and frozen in liquid nitrogen. The frozen brain tissue was ground and homogenized using a Polytron homogenizer with denaturing buffer containing 7 M guanidine–HCl, 10 mM EDTA and 0.5 M Tris–HCl (pH 8.5). Proteins were extracted by sonication of the homogenized brain tissue in ice-cold lysis buffer containing 50 mM ammonium bicarbonate (pH 8.2) and 8 M urea. After centrifugation at 12 000 g for 15 min at 10 °C, the supernatant was recovered and the concentration of the obtained protein extracts was determined with Bradford assay.

### Glycoprotein/glycopeptide enrichment

Generally, 10 mM sodium periodate was added to 100 μg of protein sample to oxidize the diols of the glycoproteins to aldehydes. The sample was incubated at 4 °C in the dark for 0.5 h. After desalting, the oxidized sample was incubated with 0.1 mg of poly-(AA-*co*-hydrazide) in 100 μL 25 mM ammonium bicarbonate (pH 6) for 1 hour with agitation to allow aldehydes–hydrazide coupling. Next, 1% TFA was added to lower the pH to ≤2 to induce large scale self-assembly and precipitation of the poly-(AA-*co*-hydrazide)–glycoprotein conjugates. The precipitated conjugates were recovered by gentle centrifugation using a micro-centrifuge. After removing the supernatant, 1% NH_3_OH was added to raise the pH to ≥6 and to re-dissolve the precipitates with gentle agitation. The re-dissolved conjugates were washed three times to remove the non-specifically adsorbed non-glycoproteins by 200 μL 50% ACN containing 8 M urea and 1 M NaCl (pH 6) using the same low-pH-precipitation and high-pH-dissolution cycle for conjugates recovery. For the kinetics study and conversion rate determination of the glycoprotein enrichment reaction, the glycoprotein (RNase B) remaining in the supernatant after 5, 10, 30, 60, 120, 240, 360 and 480 min of coupling with poly-(AA-*co*-hydrazide) was quantified by measuring its UV adsorption at 280 nm using a NanoDrop 2000c UV-Vis Spectrophotometer (Thermo Fisher Scientific, Waltham, MA, USA). For recovery evaluation of glycoprotein enrichment using asialofetuin, the enriched poly-(AA-*co*-hydrazide)–asialofetuin conjugates were dissolved in 50 μL 25 mM ammonium bicarbonate (pH 8.0) containing 100 unit of PNGase F and incubated at 37 °C overnight to release the glycoprotein. The released asialofetuin was analysed and quantified by SDS-PAGE and the results were compared with that of asialofetuin without enrichment. For glycopeptide analysis, the poly-(AA-*co*-hydrazide)–glycoprotein conjugates were dissolved in 25 μL 25 mM ammonium bicarbonate containing 8 M urea for denaturation followed by DTT reduction and IAA alkylation. After diluting the solution with 25 mM ammonium bicarbonate to reduce the urea concentration below 1 M, trypsin was introduced at a protein to trypsin ratios of 25 : 1 and the mixture was incubated at 37 °C for 16 h to digest the proteins into peptides. The non-glycopeptides generated by trypsin digestion were removed by repeated washing with 200 μL 50% ACN containing 8 M urea and 1 M NaCl (pH 6) using the same low-pH-precipitation and high-pH-dissolution cycle. The obtained poly-(AA-*co*-hydrazide)–glycopeptide conjugates were dissolved in 50 μL 25 mM ammonium bicarbonate (pH 8.0) containing PNGase F (100 unit) and incubated at 37 °C overnight to release the glycopeptides. The obtained glycopeptides were freeze-dried and 1/3 of the re-dispersed sample was used for each LC-MS analysis.

### MALDI-TOF-MS analysis

The enriched *N*-glycopeptides were re-dispersed in 5 μL CHCA solution (5 mg mL^–1^, 50% ACN, 0.1% TFA) and 1 μL of sample was spotted on the target plate and air dried. MALDI-TOF-MS analysis was performed using a 4800 MALDI-TOF-TOF analyzer (AB Sciex, USA) equipped with a Nd:YAG laser at excitation wavelength 355 nm. All the mass spectra (1000 laser shots for every spectrum) were acquired in positive reflection mode and analysed by Data Explorer (Version 4.5).

### LC-MS analysis and data processing

The LC-MS/MS analysis was carried out on an Agilent 1100 nanoLC system coupled with a hybrid linear ion trap-7 T Fourier transform ion cyclotron resonance mass spectrometer (LTQ-FT MS). The spray voltage was set to 1.8 kV. All of the MS and MS^2^ spectra were acquired in the data-dependent mode and the mass spectrometer was set to a full scan MS followed by ten data-dependent MS/MS scans. For data processing, all of the MS/MS spectra were searched against the UniProt database (version 201204206, 65 493 entries) using Protein Discoverer software (version 1.3, Thermo Scientific). Trypsin was chosen as the proteolytic enzyme and up to two missed cleavages were allowed. Carbamidomethyl (Cys) was set as the fixed modification and oxidation (Met) was set as the variable modification. The mass tolerance of the precursor ion was set to 20 ppm, that of the fragment ions was set to 0.8 Da and the peptide false discovery rate (FDR) was set to 1%. The localization of the *N*-glycosylation sites of the glycopeptides was determined by a mass shift of 0.984 Da on the N-X-S/T (X*P) sequon after deamidation of the asparagine residue into aspartic acid by PNGase F de-glycosylation.
