# Supplementary material for: A pH-responsive soluble polymer-based homogeneous system for fast and highly efficient N-glycoprotein/glycopeptide enrichment and identification by mass spectrometry
Source: Chem Sci. 2015 May 26;6(7):4234–41. doi: 10.1039/c5sc00396b (PMC5707513; doi:10.1039/c5sc00396b)
Supplement: Supplementary file 2 [file SC-006-C5SC00396B-s002.pdf]

Electronic Supplementary Material (ESI) for Chemical Science.
This journal is © The Royal Society of Chemistry 2015

A pH-responsive Soluble Polymer-based Homogeneous System for Fast and Highly Efficient N-glycoprotein/glycopeptide Enrichment and Identification by Mass Spectrometry

Haihong Bai,[§ab] Chao Fan,[§a] Wanjun Zhang,[§a] Yiting Pan,[ab] Lin Ma,[c] Wantao Ying[a] Jianhua Wang,[c] Yulin Deng,[b] Xiaohong Qian*[a] and Weijie Qin*[a]

[a] National Center for Protein Sciences Beijing, State Key Laboratory of Proteomics, Beijing Proteome Research Center, Beijing Institute of Radiation Medicine, Beijing, China.

[b] School of Life Science and Technology, Beijing Institute of Technology, Beijing, China.

[c] Research Center for Analytical Sciences, College of Sciences, Northeastern University, Shenyang, China.

* Corresponding authors' E-mail: aunp_dna@126.com and qianxh1@163.com

§ These authors contributed equally to this work.

The molecular weight and pH response of poly-(AA-co-MA), the standard curve of BCA titration of poly-(AA-co-hydrazide) copolymer, UV adsorption analysis of the pH-dependent transparency changes of poly-(AA-co-hydrazide)-asialofetuin conjugates, SDS-PAGE characterization of glycoprotein recovery of poly-(AA-co-hydrazide) based enrichment, the number of glycopeptides and non- glycopeptides identified using different methods were included in the supporting information.

**Polymerization time**	1h	2h	4h	10h	20h
**Mn (g/mol)**	15440	36330	63430	144900	214100
**Mw/Mn**	1.205	1.702	1.183	1.492	1.662

Table S1. Molecular weight of poly-(AA-co-MA) obtained using different polymerization time.

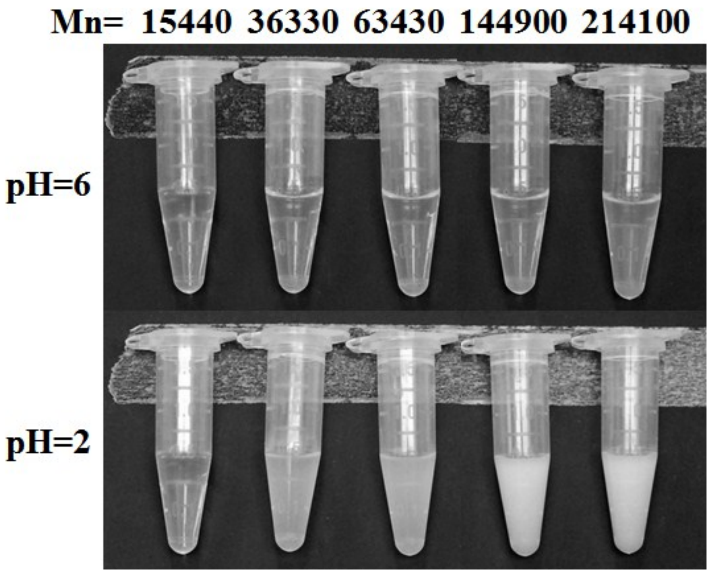

Figure S1. pH response of poly-(AA-co-MA) with molecular weights ranging from 15440 to 214100.

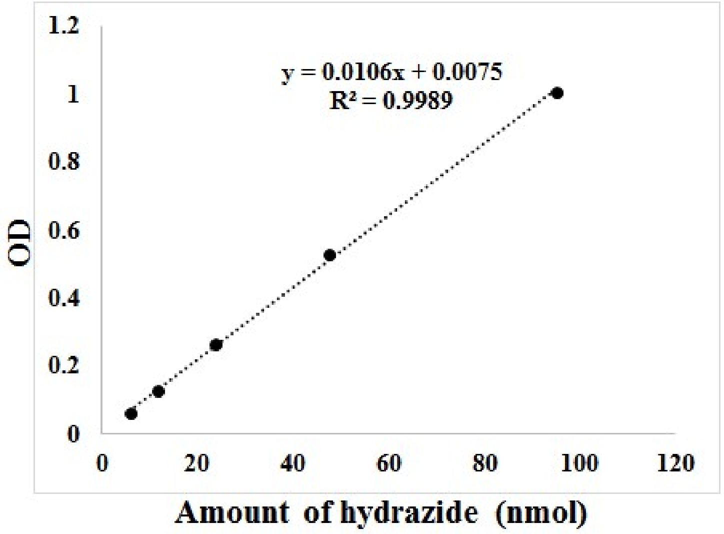

Figure S2. Standard curve of BCA titration of poly-(AA-co-hydrazide) copolymer.

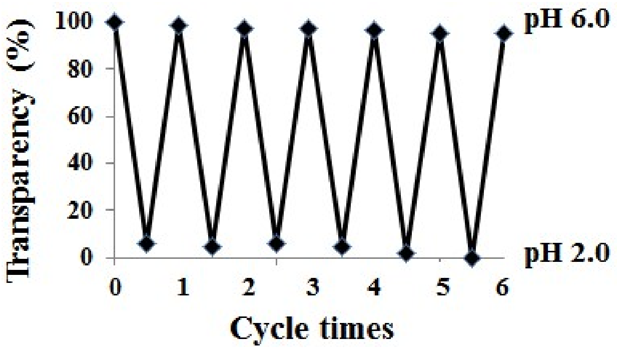

Figure S3. UV absorption analysis of the pH-dependent transparency changes of the poly-(AA-co-hydrazide)-asialofetuin conjugates. The UV absorption of the poly-(AA-co-hydrazide)-asialofetuin conjugates at pH 6.0 before the first cycle was set as 100% transparency and the UV adsorption of the conjugates at pH 2.0 in the sixth cycle was set as 0% transparency.

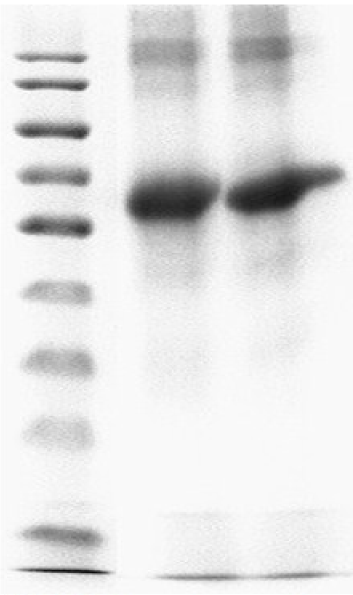

Figure S4. SDS-PAGE characterization of poly-(AA-co-hydrazide)-asialofetuin conjugates (lane 1) and free asialofetuin (lane 2) after PNGase F treatment.

**Poly-(AA-co-hydrazide)**				
	Test 1	Test 2	Test 3	Total non-redundant
N-glycopeptides	843	748	965	1317
Non-glycopeptides	429	372	472	741
**Commercial cross-linked agarose-hydrazide beads**				
	Test 1	Test 2	Test 3	Total non-redundant
N-glycopeptides	300	321	286	533
Non-glycopeptides	1128	1136	1359	2135
**Non-enriched**				
	**Test 1**	**Test 2**	**Test 3**	Total non-redundant
N-glycopeptides	29	23	26	56

Table S3. The number of identified N-glycopeptides and non-glycopeptides obtained by different methods.
